# Analysis of the health effects of multiple social networks on the older adult: the substitution role of labor participation

**DOI:** 10.3389/fpubh.2024.1501597

**Published:** 2024-12-02

**Authors:** Zhiying Li

**Affiliations:** School of Government, University of Chinese Academy of Social Sciences, Beijing, China

**Keywords:** social networks, weak ties, older adult, health effects, labor participation

## Abstract

**Objectives:**

This study aims to examine the role of social networks in influencing the physical and mental health of older Chinese adults, investigating both the underlying mechanisms and the associations between social networks, labor force participation, and health outcomes.

**Methods:**

Using data from the 2021 China General Social Survey (CGSS), we analyzed a sample of 1,332 older adults, incorporating demographic and health-related variables. Ordinary least squares (OLS) regression and mediation analysis were conducted to evaluate the effects of social networks on health outcomes, with subgroup analyses by gender and education level. Stata 18.0 and SPSS were employed to perform all statistical analyses.

**Results:**

Social networks demonstrate a significant positive effect on overall, physical, and mental health at the 1% level, with weak-tie networks exhibiting a more substantial impact on health (*β* = 0.1146, *p* < 0.01). In promoting physical health, social networks have a coefficient of 0.1371 (*p* < 0.001) for females and 0.2128 (*p* < 0.001) for males. Among individuals with lower education, the coefficient is 0.1561 (*p* < 0.001), while for those with higher education, it is 0.2184 (*p* < 0.001). Regarding mental health, social networks yield a coefficient of 0.0747 (*p* > 0.05) for females and 0.1095 (*p* < 0.01) for males; for individuals with lower education, the coefficient is 0.0914 (*p* < 0.01), and for those with higher education, it is 0.0441 (*p* > 0.05). Media use, subjective wellbeing, and perceived social class are key explanatory mechanisms in the relationship between social networks and health of the older adult. Notably, subjective wellbeing and perceived social class function as chain mediators between social networks and health outcomes. The interaction between social networks and labor participation reveals a significant negative coefficient (*β* = −0.1864, *p* < 0.01).

**Conclusion:**

Social networks contribute to improved health in older adults, with weak ties playing a particularly significant role, although the effect varies across subgroups. Media use, subjective social class, and wellbeing are important mechanisms linking social networks with older adult health, while labor force participation may serve as a substitute for social networks in health promotion among older adults. This study will inform the improvement of older adults health and the development of labor policies for older adults.

## 1 Introduction

China is facing a significant challenge of rapid population aging, characterized by a large scale, fast pace, and the distinct feature of “growing old before getting rich.” By the end of 2023, the population aged 60 and above had reached 296.97 million (up from 280.04 million in 2022), accounting for 21.1% of the total population (19.8% in 2022). Among them, 216.76 million people were aged 65 and above (up from 209.78 million in 2022), comprising 15.4% of the population (14.9% in 2022). The older adult population is expected to continue growing, reaching a peak of 398 million by 2060. A comparison of data from the sixth and seventh national censuses reveals that although there has been some improvement in the health of the older adult population in recent years, significant health disparities remain between urban and rural older adult. As life expectancy increases, the proportion of older adult individuals and the number of disabled older adult will continue to rise, further intensifying the demand for older adult care, healthcare, and other social services.

In recent years, China has implemented several policy measures to support the development of older adult human resources. The Central Committee of the Communist Party of China and the State Council have successively issued the “National Medium and Long-Term Plan for Actively Responding to Population Aging” and the “Opinions on Strengthening Older Adult Work in the New Era,” which clarify key areas such as employment, volunteer services, and community governance, aiming to fully utilize the potential of the older adult population and actively explore flexible employment models suitable for them. The “14th Five-Year Plan” and the “Outline of Long-term Goals for 2035” propose a gradual delay of the statutory retirement age, signaling the planned implementation of the delayed retirement policy in China. While numerous studies have focused on the impact of labor participation on older adult health, the conclusions remain inconsistent. From the perspective of activity theory, older adult individuals gain sustained activity levels and social interaction through labor participation, which helps them achieve self-realization and improve daily living abilities (1, 2). However, from the perspective of stress theory, the pressure of work can lead to emotional tension among older adult workers, harming their physical health and increasing mortality risk (3).

Based on previous research, this study utilizes the latest large-scale microdata (CGSS2021) to empirically investigate the mechanisms by which social networks influence the health of older adults. The population of this study was older adults, with an intervention group of older adults with greater social network density and comparison group of older adults with low social network density, and the outcome was their health level. The primary objective is to address the question “Do social networks affect the health of older adults?”; the secondary objective is to explore the specific mechanisms by which social networks affect the health of older adults and to analyze what role labor participation plays in the promotion of older adults’ health by social networks. This study aims to provide feasible policy recommendations for improving the health of the older adult, and to explore realistic paths for the re-employment of the older adult, guided by the theory of health promotion, in order to positively respond to the challenges posed by population aging.

## 2 Literature review

Social networks refer to the relatively stable systems of relationships formed by individuals through interactions with other social members (4). These networks can be divided into strong and weak ties, which are measured by the frequency of interaction, relationship type, importance, and intimacy (5). In terms of individual characteristics, strong ties exist within groups of individuals with similar traits, resulting in smaller, more stable networks where information tends to be more homogeneous. In contrast, weak ties transmit more heterogeneous information and serve as channels for information exchange (6). In terms of social class, strong ties exist between individuals of similar social standing, whereas weak ties span different social classes with disparate resources (7). From a spatial perspective, strong ties exist among individuals in close proximity, who interact more frequently, whereas distant groups interact less frequently (8).

As a critical microenvironment, social networks influence daily life and physical and mental health (9). Extensive research shows that social networks help individuals access more information and resources, fulfill basic mental needs, reduce depression risk (10), and positively affect health outcomes (11–15). However, some scholars argue that the positive impact of social networks on health is only effective for low-income groups (16). Others believe social networks can exacerbate social comparisons and relative deprivation, transmitting negative emotions that harm both physical and mental health (17). Subgroup studies show that older adult women tend to have larger social networks (18), and cognitive functions within social networks can protect against dementia progression in women (19).

There is also extensive debate about the effects of older adult labor participation on health, with no consensus. Some scholars argue that labor participation negatively impacts health, increasing the risk of obesity among long-term workers (20, 21) and raising the likelihood of chronic diseases (22). Conversely, others believe that labor participation positively impacts health by helping older adult individuals maintain healthy routines, fully utilizing their physical and intellectual potential (23), and enhancing their sense of self-worth and wellbeing through empowerment (24). This fulfillment of social and psychological needs promotes overall physical and mental health. Some researchers suggest that the health effects of labor participation depend on the relationship between the marginal costs of health investments and the marginal benefits of health consumption (25), with moderate work promoting health, while high-intensity and long-term work can harm it.

While existing research has made substantial progress, some unavoidable issues remain. First, the terms “social network,” “social capital,” and “social relations” are often used interchangeably, leading to a lack of clarity in defining social networks. Second, current research on older adult health has not been sufficiently thorough, focusing primarily on demographic factors like gender, education, living arrangements, and marital status, as well as daily activities like caregiving and leisure, and economic factors like intergenerational support and financial security. There is a notable lack of research on social networks and how labor participation mediates their impact on older adult health. Third, empirical studies on older adult health tend to use narrow measures, often relying solely on “self-rated health,” with limited multidimensional considerations or detailed analysis of representative variables within each dimension.

## 3 Theoretical analysis and hypotheses

Activity theory posits that maintaining consistent activity levels and social interactions is crucial for enhancing wellbeing and achieving healthy aging. Participation in social interactions by older adults can delay the aging process and improve their quality of life (26). As a key microenvironment, social networks significantly influence individuals’ daily lives and physical and mental health (9, 27). Existing research indicates that social networks are an important reference for analyzing older adult health; older adults with denser social networks tend to exhibit better health outcomes compared to those with less dense networks. According to the theory of strong and weak ties, strong ties involve individuals with similar knowledge, experience, and backgrounds, which leads to high redundancy of information, providing little new insight. In contrast, weak ties serve as bridges between different social circles and networks, allowing older adults to interact with people of diverse knowledge structures and experiences, thus gaining access to more external information (6, 28). This exposure to diverse information and broader perspectives is beneficial for older adult health. Based on this, Hypothesis 1 is proposed:

*H1*: Social networks enhance the health of older adults, with weak ties playing a more significant role than strong ties.

Older people with a high density of social networks also have a higher rate of media use, and the high frequency of media use is conducive to older people acquiring health knowledge from a variety of sources, enhancing their health and sense of wellbeing. Subjective social class refers to an individual’s perception of their own social status, which is primarily shaped through social interactions. Objective economic conditions alone cannot fully explain this perception, as the social environment influences individuals’ judgments of their subjective social class (29). An individual’s class identity is influenced by other members within their social network, which can positively reinforce their sense of class identity. The higher an individual’s subjective class identification, the lower their negative emotions, thereby contributing to improved health among older adults. Furthermore, numerous studies have found a significant positive correlation between social networks and subjective wellbeing (53–55). There is also a strong link between wellbeing and health (30, 31). Specifically, lower wellbeing can lead to mental health issues, which in turn trigger health-damaging behaviors (32), further affecting physical health. Conversely, individuals with higher wellbeing are more likely to adopt healthier dietary habits, engage in physical exercise (33–35), and pursue a healthier lifestyle, thereby contributing to better health outcomes. Based on this, Hypothesis 2 is proposed:

*H2*: Media use, subjective social class, and wellbeing mediate the relationship between social networks and the health of older adults.

After retirement, older adults experience various changes in their living environment, including shifts in social roles, psychological states, cognition, interpersonal relationships, and social networks. According to Maslow’s hierarchy of needs theory (36), while basic needs of older adults may be met, their higher-level needs, such as self-actualization, often remain unmet. In the long term, ceasing labor participation can negatively affect both their mental and physical health. Resocialization theory suggests that older adults, through resocialization, can manage their leisure time more effectively, break out of closed interpersonal networks, and actively engage in social life. By participating in labor in a rational manner, they can better realize self-worth and create social value. This is particularly true for older adults with less dense social networks, for whom labor participation can substitute for social networks in promoting health. Based on this, Hypothesis 3 is proposed:

*H3*: Labor participation among older adults can substitute for social networks in promoting their health.

## 4 Materials and methods

### 4.1 Data

The data for this study came from the 2021 China General Social Survey (CGSS) conducted from May to September 2021 by the China Survey and Data Center of Renmin University of China. CGSS is a nationwide, comprehensive, and continuous academic survey program implemented since 2003, using the multistage stratified PPS method for random sampling, with a sample covering 28 provinces in China (excluding Xinjiang, Tibet, Hainan, and Hong Kong, Macao, and Taiwan). The dataset systematically and comprehensively collects data at multiple levels, such as the community, the household, and the individual, and with a large sample size and strong representativeness, it has become one of the most important sources of data for the study of contemporary The dataset systematically and comprehensively collects data at multiple levels, such as community, household and individual, with a large sample size and strong representativeness, and has become one of the most important data sources for studying contemporary Chinese society. The study focuses on individuals aged 60 and above. After excluding the samples with missing values for the variables of interest, 1,336 valid samples were obtained.

### 4.2 Variables

#### 4.2.1 Dependent variables

The dependent variable in this study is the health level of older adults, which includes both physical and mental health. Physical health is measured by the question “How is your current physical health?” and mental health is measured by “How often do you feel depressed or down?” Both questions are scored on a scale of 1–5, with higher scores indicating better health in the respective dimension. An average of the scores for physical and mental health is taken to construct a composite health index, ranging from 1 to 5, where higher values represent better overall health.

#### 4.2.2 Independent variables

The independent variable is the social networks. According to the theory of strong and weak ties (6), the strength of social networks ties can vary based on emotional intensity, reciprocity, intimacy, and frequency of contact. Strong ties typically occur in more homogenous and tightly-knit groups, characterized by long-term, stable relationships, commonly seen among relatives, close friends, and other strong bonds (37). In this study, strong ties are measured using three indicators from the questionnaire: “frequency of gatherings with relatives,” “frequency of gatherings with friends,” and “frequency of social and recreational activities with friends.” In this study, the scores of the above three indicators were averaged to form the strong ties indicator. Weak ties, on the other hand, are found in groups with lower social overlap, less time investment, and lower frequency of communication and contact, such as neighbors, acquaintances, or vendors. Weak ties provide older adults with access to people with different knowledge structures and backgrounds, offering more diverse external information (6, 28). In this study, weak ties are measured using four indicators: “frequency of shopping,” “frequency of exercise,” “frequency of cultural activities,” and “frequency of social and recreational activities with neighbors.” In this study, the scores of the above four indicators were averaged to form the weak ties indicator.

A Likert scale of 1–5 was used for both strong and weak ties, with 1 representing the frequency of that social network as “never,” 2 representing “a few times a year or less,” 3 representing “a few times a month,” 4 for “a few times a week,” and 5 for “every day.” The average of the two dimensions, strong and weak ties, was used to construct a social network index ranging from 1 to 5, with higher values indicating greater social network density.

#### 4.2.3 Mediating variables

The mediating variables include media usage frequency, subjective social class, and wellbeing. Subjective social class is measured by the question “Which social class do you think you belong to?” on a scale of 1–10, with higher scores indicating higher social class identification. Wellbeing is measured by the question “Do you feel happy with your life?” on a scale of 1–5, with higher scores indicating greater wellbeing. Media usage frequency is measured by the question “How frequently do you use the following media?” and includes six dimensions: newspapers, magazines, radio, television, the internet (including mobile internet), and mobile message subscriptions. An average score is taken, ranging from 1 to 5, with higher scores indicating more frequent media usage.

#### 4.2.4 Moderating variables

The moderating variable is labor participation, measured by the question “What is your employment status?” Responses are categorized into two groups: those who are not currently participating in labor (combining “never worked,” “currently unemployed but previously engaged in non-agricultural work,” and “currently unemployed and only engaged in agricultural work”) are assigned a value of 0, while those who are currently participating in labor (combining “currently employed in non-agricultural work,” “currently engaged in agriculture but previously engaged in non-agricultural work,” and “currently engaged in agriculture without prior non-agricultural work”) are assigned a value of 1.

#### 4.2.5 Control variables

To minimize the influence of other factors on the health of older adults, this study includes a set of control variables categorized into three groups: basic personal characteristics, human capital characteristics, and family characteristics. The control variables for personal characteristics include gender, age, ethnicity, religion, and household registration status. The human capital characteristics include education level, political affiliation, income, and participation in insurance. Family characteristics include living arrangements and the number of children.

### 4.3 Econometric model

In this study, Stata 18.0 was used and multiple linear regression model was employed to estimate the effect of social networking on health of older adults. Results were considered statistically significant when they rejected Alpha at *p* < 0.05. The model is expressed as follows:

(1) $$\mathrm{Heal}th_{i}=\alpha_{i}+\beta_{1i}\mathrm{Networ}k_{i}+\overset{k}{\underset{j=1}{\sum }}\beta_{2i}X_{ij}+\varepsilon_{i}$$

In Equation 1, $\mathrm{Healt}h_{i}$ represents the health level of individual i, $\mathrm{Networ}k_{i}$ denotes the social networks of individual i, $X_{ij}$ refers to the control variables, including personal characteristics, human capital features, and family attributes as detailed in Table 1, $\alpha_{i}$ is the intercept term, $\beta_{1i}$ is the regression coefficient of the independent variable $\mathrm{Networ}k_{i}$ , $\beta_{2i}$ are the regression coefficients of the control variables, j is the number of control variables, and $\varepsilon_{i}$ represents the random disturbance term.

**Table 1:** Descriptive statistics of major variables (*N* = 1,332).

Variable type	Variable name	Mean	SD	Min	Max
Dependent variables	Health	3.519	0.942	1	5
	Physical health	3.145	1.102	1	5
	Mental health	3.893	1.124	1	5
Key independent variables	Social networks	2.443	0.827	1	4.542
	Strong ties	2.28	0.95	1	4.75
	Weak ties	2.607	0.926	1	4.833
Mediating variables	Subjective social class	4.361	1.973	1	10
	Media use	2.146	0.678	1	4.5
	Subjective wellbeing	4.078	0.805	1	5
Moderating variables	Labor participation	0.254	0.436	0	1
Personal characteristics	Gender	0.501	0.5	0	1
	Age	69.947	6.911	60	95
	Ethnicity	0.944	0.23	0	1
	Religion	0.08	0.272	0	1
	Hukou (household registration)	0.352	0.478	0	1
Human capital characteristics	Education	2.613	1.191	1	7
	Political affiliation	0.19	0.393	0	1
	Income	24649.778	30779.384	0	360,000
	Insurance	0.967	0.179	0	1
Family characteristics	Living arrangement	0.631	0.483	0	1
	Number of children	2.219	1.275	0	12

To explore potential complementary or substitutive effects between labor participation and social networks, the following multiple regression model is constructed:

(2) $$\begin{array}{l} \mathrm{Healt}h_{i}=\\ \alpha_{i}+\beta_{1i}\mathrm{Networ}k_{i}+\beta_{1i}\mathrm{Networ}k_{i}\times \mathrm{Labo}r_{i}+\overset{k}{\underset{j=1}{\sum }}\beta_{2i}X_{ij}+\varepsilon_{i} \end{array}$$

In Equation 2, $\beta_{1i}\mathrm{Networ}k_{i}\times \mathrm{Labo}r_{i}$ represents the interaction term between social networks and labor participation for individual i, used to examine whether labor participation moderates the effect of social networks on the health of older adults.

**Figure 1:**
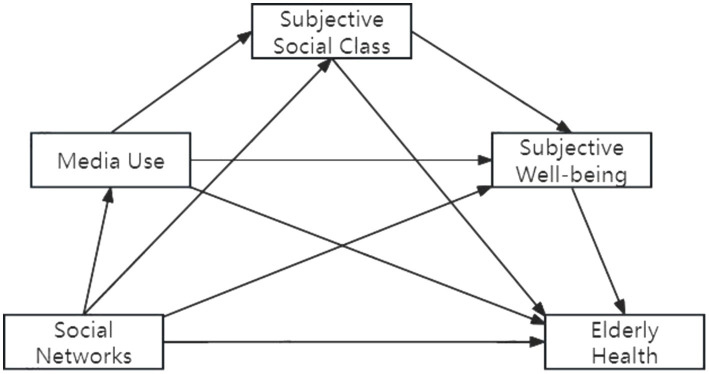
Chain mediation effect testing model.

To test the chain mediation effects of media usage, subjective social class, and wellbeing between social networks and older adults’ health, this study employs the SPSS Process 3.5 plugin, using Model 6 (chain mediation model). The bootstrap method is applied with 5,000 resamples to construct a 95% bias-corrected confidence interval for testing the significance of the chain mediation effects (Figure 1).

This study employs OLS (Ordinary Least Squares) estimation to examine the health of older adults. Typically, OLS estimates are persuasive only when multicollinearity is absent. Therefore, it is essential to rule out the effects of multicollinearity. The variance inflation factor (VIF) of the variables is all below 10, with an overall VIF coefficient of 1.28, indicating that there are no severe multicollinearity issues in the model.

## 5 Results

### 5.1 Full analysis

After standardizing the main variables, an OLS regression model is used to assess the health of older adults (both physical and mental) as the dependent variables, with social networks as the core independent variable. The results indicate that social networks have a significantly positive effect on overall health, physical health, and mental health at the 1% level (Table 2).

**Table 2:** OLS regression results of the impact of social networks on older adult health.

Variable name	Overall health	Physical health	Mental health
	Model 1	Model 2	Model 3
Social networks	0.1320^***^	0.1771^***^	0.0869^***^
	(0.0270)	(0.0329)	(0.0323)
Gender	0.1338^***^	0.0895	0.1781^***^
	(0.0491)	(0.0599)	(0.0589)
Age	−0.0019	−0.0099^**^	0.0062
	(0.0039)	(0.0048)	(0.0047)
Ethnicity	0.0719	0.0217	0.1222
	(0.1103)	(0.1345)	(0.1322)
Religion	0.0406	0.0737	0.0075
	(0.0884)	(0.1078)	(0.1059)
Hukou (household registration)	0.0977	0.0846	0.1107
	(0.0598)	(0.0730)	(0.0717)
Education	0.0455^*^	0.0316	0.0594^**^
	(0.0244)	(0.0297)	(0.0292)
Political affiliation	0.1514^**^	0.1159	0.1870^**^
	(0.0620)	(0.0757)	(0.0743)
Income	0.0928^***^	0.1020^***^	0.0837^***^
	(0.0214)	(0.0261)	(0.0257)
Insurance	−0.0543	0.0411	−0.1496
	(0.1424)	(0.1736)	(0.1706)
Labor participation	0.1366^**^	0.1496^**^	0.1235^*^
	(0.0582)	(0.0710)	(0.0697)
Living arrangement	0.0028	−0.0371	0.0428
	(0.0492)	(0.0601)	(0.0590)
Number of children	−0.0363^*^	−0.0256	−0.0471^*^
	(0.0211)	(0.0257)	(0.0253)
_cons	−0.9271^**^	−0.4159	−1.4383^***^
	(0.3676)	(0.4483)	(0.4404)
Adj. *R*^2^	0.1158	0.0831	0.0875

^*^p < 0.1, ^**^p < 0.05, ^***^p < 0.01.

Table 3 presents the OLS regression results for the impact of multiple social networks on the health of older adult individuals, controlling for other variables. The findings indicate that for each one unit increase in strong tie networks, the overall health of older adult individuals improves by 0.0887 units. In contrast, the effect of weak tie networks on the overall health of the older adult is more pronounced, with a coefficient of 0.115.

**Table 3:** OLS regression results for multiple social networks on older adult health.

Variable name	Overall health		Physical health		Mental health	
	Model 1	Model 2	Model 3	Model 4	Model 5	Model 6
Strong ties	0.0887^***^		0.1215^***^		0.0560^**^	
	(0.0238)		(0.0291)		(0.0284)	
Weak ties		0.1146^***^		0.1514^***^		0.0778^***^
		(0.0236)		(0.0288)		(0.0283)
Control variables	Yes	Yes	Yes	Yes	Yes	Yes
_cons	−0.9194^**^	−0.9801^***^	−0.4032	−0.4876	−1.4355^***^	−1.4727^***^
	(0.3696)	(0.3673)	(0.4510)	(0.4482)	(0.4415)	(0.4400)
Adj. *R*^2^	0.1079	0.1154	0.0737	0.0819	0.0848	0.0878

^*^p < 0.1, ^**^p < 0.05, ^***^p < 0.01.

### 5.2 Endogeneity treatment and robustness analysis

#### 5.2.1 Discussion and treatment of endogeneity

There may be potential endogeneity issues in the model, particularly due to omitted variable bias and reverse causality. Omitted variables, such as unobservable individual characteristics, could affect both social networks and health levels. For instance, pessimistic respondents may subjectively undervalue their social network density, which might also reflect lower health outcomes. Additionally, health levels may also influence social networks. To address this, this study draws on Wei et al. (38) practice of using the average of the social networks of other individuals within the same village residence as an instrumental variable for the following reasons: first, to satisfy the correlation hypothesis. There are a large number of clan relations and acquaintance culture in the same village, and an individual’s social network largely originates from the interaction with the people around him or her, which leads to the convergence of an individual’s social network with other people in the village, and the two have a strong positive correlation; and secondly, it satisfies the exogenous hypothesis that the social networks of other people in the same village do not have a direct effect on the health of an individual. Therefore, it is feasible to select other individual social networks within the same village residence as instrumental variables, which basically fulfill the requirements of relevance and exogeneity.

The results from the two-stage least squares (2SLS) regression show that the social network instrument passes the weak instrument test (Kleibergen-Paap rk Wald F-statistic = 15.85, above the critical value of 8.96), confirming its validity (Table 4).

**Table 4:** Two-stage regression results with instrumental variables.

Variable name	First stage	Second stage
	Social networks	Health levels
Instrument	0.204^***^	
	(0.051)	
Social networks		0.560^**^
		(0.251)
Control variables	Yes	Yes
_cons	−0.432	−0.699
	(0.410)	(0.429)
Observations	1,105	1,105
*R*^2^	0.051	/

^*^p < 0.1, ^**^p < 0.05, ^***^p < 0.01.

In the first stage of the 2SLS analysis, the correlation coefficient between individual social networks and the average social networks of others in the region is 0.204, confirming a positive influence of regional average social networks on individual social networks. This relationship is significant at the 1% level. The Kleibergen-Paap rk Wald F-statistic is 15.85, exceeding the 10%bias critical value of 8.96, thereby passing the weak instrument variable test. Thus, the average social network of others in the village or neighborhood is a strong and valid instrument variable.

#### 5.2.2 Robustness check

To ensure the reliability of the empirical results, the study conducts robustness checks using propensity score matching (PSM). Four matching methods are applied—nearest neighbor matching, radius matching, kernel matching, and local linear regression matching. In all cases, the average treatment effect of social networks on older adult health is significantly positive at the 1% level. Balance tests indicate that most variables have standardized bias below 10%, and t-test results are insignificant at the 1% level, confirming the absence of systematic differences between the treatment and control groups (Table 5).

**Table 5:** Propensity score matching results based on different methods.

Matching method	ATT	SD	*t*-value	Treated observations	Control observations
Nearest neighbor matching	0.2141	0.0539	3.98	569	555
Radius matching	0.1902	0.0499	3.81	567	552
Kernel matching	0.2002	0.0492	4.06	569	559
Local linear regression	0.2104	0.0649	3.24	569	559

Additionally, a robustness check was performed using variable substitution and different empirical methods. The results indicate that social networks remain significantly positively correlated with older adult health, demonstrating that the empirical findings are robust and the conclusions drawn from them are reliable.

### 5.3 Mechanism analysis

#### 5.3.1 Mediation effect

This study examines the chain mediation effect of media usage, subjective social class, and subjective wellbeing, using social networks as the independent variable and older adult physical and mental health as the dependent variables.

**Figure 2:**
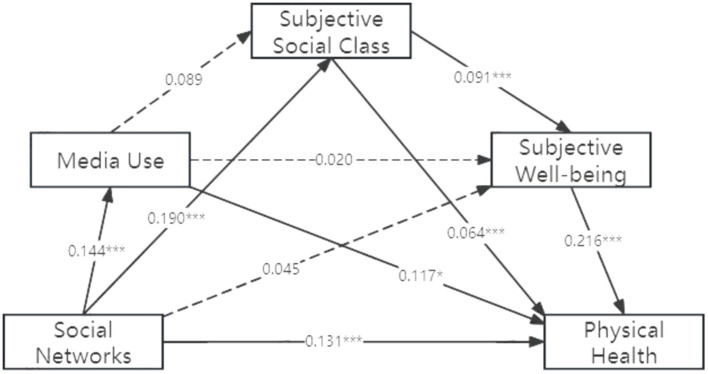
Chain mediation model of social networks’ impact on older adult physical health.

The results show that the impact of social networks on physical health among the older adult includes a mediation effect. Social networks significantly predict media usage (*β* = 0.144, *p* < 0.001), subjective social class (*β* = 0.190, *p* < 0.01), and physical health (*β* = 0.131, *p* < 0.001). Subjective social class significantly predicts subjective wellbeing (*β* = 0.091, *p* < 0.001) and physical health (*β* = 0.064, *p* < 0.001), while subjective wellbeing significantly predicts physical health (*β* = 0.216, *p* < 0.001) (Figure 2).

**Figure 3:**
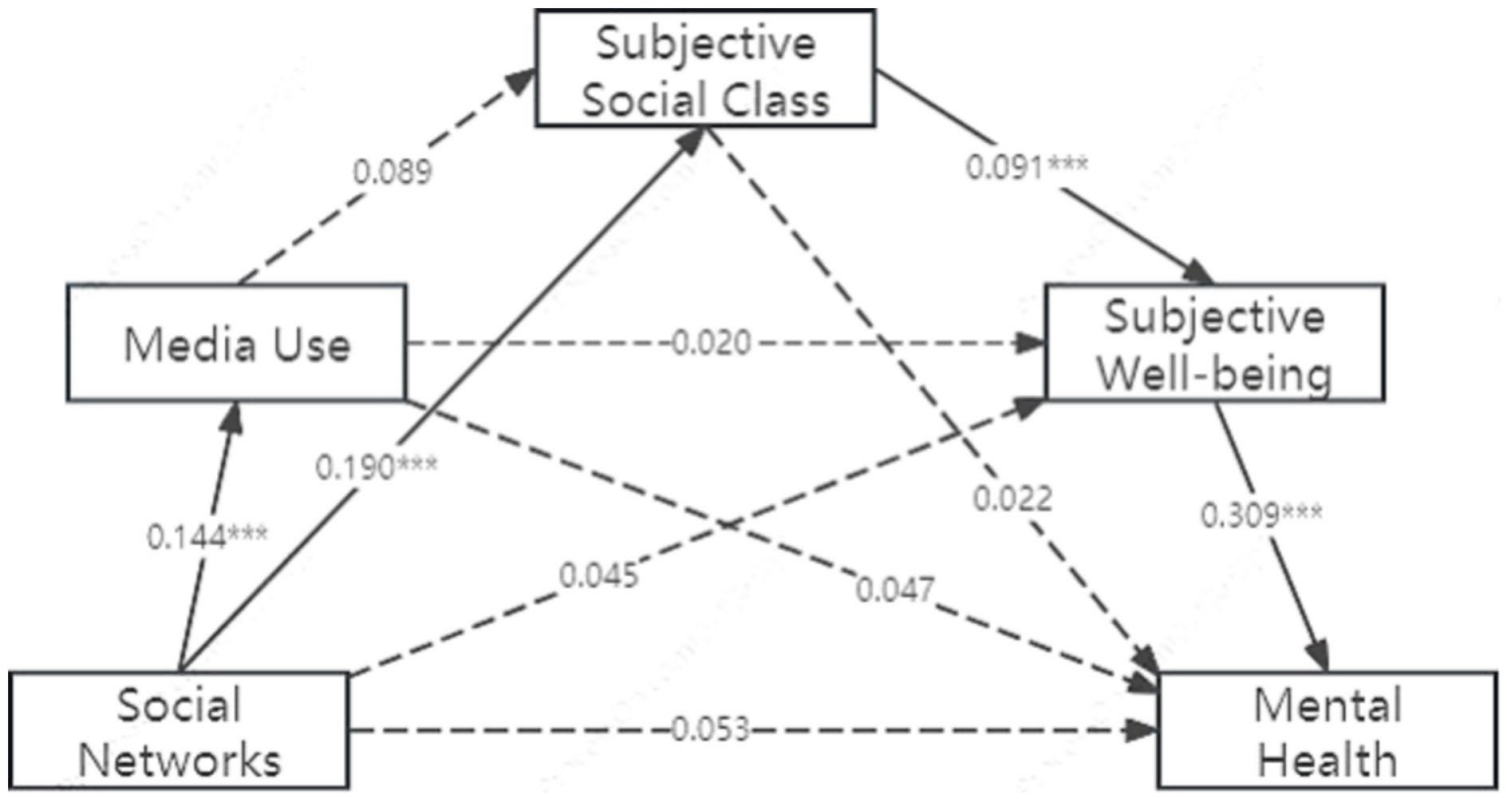
Chain mediation model of social networks’ impact on older adult mental health.

The chain mediation effect also exists between social networks and mental health. The results show: Social Networks significantly predict Media Usage (*β* = 0.144, *p* < 0.001) and Subjective Social Class (*β* = 0.144, *p* < 0.001). Subjective Social Class significantly predicts subjective wellbeing (*β* = 0.091, *p* < 0.001). Subjective wellbeing significantly predicts Mental health (*β* = 0.309, *p* < 0.001) (Figure 3).

The study employs the bias-corrected non-parametric percentile Bootstrap method (39) to conduct a robustness check with 5,000 repetitions, calculating the 95% confidence intervals for each mediation effect. If a confidence interval does not include zero, the mediation effect is considered significant. Total Effect of Social Networks on Physical Health is 0.175 (*t* = 5.307, *p* < 0.001), while Direct Effect is 0.131 (*t* = 3.983, *p* < 0.001) and Indirect Effect is 0.044. Media usage and subjective social class serve as partial mediators between social networks and physical health, accounting for 38.64%, 27.27% of the total effect, respectively. Subjective social class and subjective wellbeing are chain mediators affecting physical health, accounting for 9.09% of the total effect (Table 6).

**Table 6:** Mediation effects analysis on physical health.

Effect type	Effect value	Standard error	Lower limit	Upper limit	Proportion (%)
Social networks → media usage → physical health	0.017	0.007	0.004	0.033	38.64
Social networks → subjective social class → physical health	0.001	0.001	−0.001	0.003	
Social networks →wellbeing → physical health	0.001	0.001	−0.002	0.003	
Social networks → subjective social class →wellbeing→ physical health	0.000	0.000	0.000	0.001	
Social networks → subjective social status → physical health	0.012	0.005	0.004	0.024	27.27
Social networks → subjective social status → wellbeing → physical health	0.004	0.002	0.001	0.008	9.09
Social networks → wellbeing →physical health	0.010	0.006	−0.001	0.024	

Total Effect of Social Networks on Mental Health is 0.085 (*t* = 2.627, *p* < 0.01), while Direct Effect is 0.053 (*t* = 1.662, *p* > 0.05) and Indirect Effect is 0.032. Subjective social class and subjective wellbeing serve as complete mediators for social networks’ impact on mental health, accounting for 16.13% of the total effect (Table 7).

**Table 7:** Mediation effects analysis on mental health.

Effect type	Effect value	Standard error	Lower limit	Upper limit	Proportion (%)
Social networks → media usage → mental health	0.007	0.007	−0.006	0.021	
Social networks → subjective social class → mental health	0.000	0.000	0.000	0.002	
Social networks →wellbeing → mental health	0.001	0.002	−0.002	0.005	
Social networks → media usage →wellbeing → mental health	0.000	0.000	0.000	0.001	
Social networks → subjective social status → mental health	0.004	0.003	0.0000	0.013	
Social networks → subjective social status → wellbeing → mental health	0.005	0.002	0.002	0.011	16.13%
Social networks → wellbeing → mental health	0.014	0.009	−0.002	0.032	

#### 5.3.2 Moderating effects

Further examination of the interaction effect between labor participation and social networks on older adult health is presented. In Model 1 of Table 8, after controlling for individual, household, and economic variables, both social networks and labor participation exhibit positive coefficients, significant at the 1 and 5% levels, respectively. However, in Model 2, the interaction term between social networks and labor participation shows a significant negative coefficient.

**Table 8:** Interaction of social networks and labor participation on overall health.

Variable name	Overall health					
	Model 1	Model 2	Model 3	Model 4	Model 5	Model 6
Social networks	0.1320^***^	0.1297^***^	0.1552^***^	0.1518^***^	0.1508^***^	0.1536^***^
	(0.0270)	(0.0269)	(0.0282)	(0.0282)	(0.0290)	(0.0289)
Labor participation	0.1366^**^	0.1238^**^				
	(0.0582)	(0.0581)				
Social networks × labor participation		−0.1864^***^				
		(0.0620)				
Agricultural labor participation			0.0489	0.0308		
			(0.0674)	(0.0679)		
Social networks × agricultural labor participation				−0.1507^**^		
				(0.0731)		
Non-agricultural labor participation					0.2854^***^	0.2881^***^
					(0.0884)	(0.0879)
Social networks × non-agricultural labor participation						−0.3072^***^
						(0.0988)
Controls	Yes	Yes	Yes	Yes	Yes	Yes
_cons	−0.9271^**^	−0.9525^***^	−0.8519^**^	−0.8492^**^	−0.7080^*^	−0.7498^*^
	(0.3676)	(0.3663)	(0.3804)	(0.3798)	(0.3887)	(0.3870)
Adj.*R*^2^	0.1158	0.1221	0.1155	0.1183	0.1365	0.1448

^*^p < 0.1, ^**^p < 0.05, ^***^p < 0.01.

Different types of labor participation have varying moderating effects on older adult health (Table 9). Notably, agricultural labor participation does not significantly impact older adult health, while both social networks and non-agricultural labor participation positively influence health at the 1% significance level.

**Table 9:** Interaction of social networks and non-agricultural labor participation on physical and mental health.

Variable name	Physical health		Mental health	
	Model 1	Model 2	Model 3	Model 4
Social networks	0.2106^***^	0.2146^***^	0.0911^***^	0.0926^***^
	(0.0352)	(0.0350)	(0.0349)	(0.0349)
Non-agricultural labor participation	0.3210^***^	0.3248^***^	0.2499^**^	0.2513^**^
	(0.1072)	(0.1065)	(0.1062)	(0.1062)
Social networks × non-agricultural labor participation		−0.4467^***^		−0.1678
		(0.1196)		(0.1193)
Controls	Yes	Yes	Yes	Yes
_cons	−0.0521	−0.1128	−1.3639^***^	−1.3867^***^
	(0.4717)	(0.4686)	(0.4673)	(0.4673)
Adj. *R*^2^	0.1078	0.1206	0.0934	0.0944

^*^p < 0.1, ^**^p < 0.05,^* **^p < 0.01.

Since agricultural labor participation does not independently impact the health of the older adult, this study focuses solely on the moderating effect of non-agricultural labor participation on the physical and mental health of older adults. The findings reveal that, in terms of the influence of social networks on older adult physical health, both the coefficients for social networks and non-agricultural labor participation are significantly positive, while the coefficient for the interaction term is significantly negative.

### 5.4 Subsample analysis

To consider the differences in gender and education levels among the older adult, a subsample regression based on these factors was conducted. The results reveal: In promoting physical health, social networks have a coefficient of 0.1371 (*p* < 0.001) for females and 0.2128 (*p* < 0.001) for males. For individuals with low education, the coefficient is 0.1561 (*p* < 0.001), and for those with high education, it is 0.2184 (*p* < 0.001). Regarding mental health, social networks have a coefficient of 0.0747 (*p* > 0.05) for females and 0.1095 (*p* < 0.01) for males, while for low-education individuals, it is 0.0914 (*p* < 0.01) and 0.0441 (*p* > 0.05) for high-education individuals (Table 10).

**Table 10:** Heterogeneity analysis of dependent variables.

Variable name	Physical health		Mental health		Physical health		Mental health	
	Female	Male	Female	Male	Low education	High education	Low education	High education
Social networks	0.1371^***^	0.2128^***^	0.0747	0.1095^**^	0.1561^***^	0.2184^***^	0.0914^**^	0.0441
	(0.0474)	(0.0463)	(0.0483)	(0.0441)	(0.0390)	(0.0614)	(0.0385)	(0.0588)
Controls	Yes	Yes	Yes	Yes	Yes	Yes	Yes	Yes
_cons	−0.9528	0.1650	−1.0126	−1.5000^**^	−0.8767	0.8378	−1.6508^***^	−0.6213
	(0.6546)	(0.6359)	(0.6663)	(0.6061)	(0.5329)	(0.8516)	(0.5262)	(0.8159)
*N*	517	611	517	611	841	287	841	287
Adj. *R*^2^	0.0777	0.0733	0.0681	0.0626	0.0626	0.0873	0.0671	0.0358

^*^p < 0.1, ^**^p < 0.05, ^***^p < 0.01.

## 6 Discussion

This study primarily explores the impact of social networks on older adult health and the underlying mechanisms. Consistent with the findings of Wilson-Genderson e al. (27), social networks have a significant positive effect on older adult health. By interacting with family, neighbors, and friends, and maintaining good communication with the outside world, older adult individuals can increase the density of their social networks. On one hand, these interactions provide opportunities to acquire health knowledge (40) and promote health-enhancing behaviors (41); on the other hand, they help alleviate feelings of loneliness (42, 43), thereby improving overall health. Heterogeneous weak ties serve as bridges within social networks (6) and help the older adult counter social disconnection. Both physical and mental health dimensions benefit more from weak tie networks.

Previous research has indicated that social networks enhance subjective wellbeing (10, 44), which holds true across various groups, including Singaporean university students (45) and migrant workers in China (46). Operario et al. (47) have also demonstrated that subjective social status can predict health outcomes. Media use and subjective social class partially mediate between social networks and older adults’ physical health, respectively; subjective social class and wellbeing are chain mediators of social networks’ influence on older adults’ physical health; and subjective social class and wellbeing are chain mediators of social networks’ influence on older adults’ mental health, and act as full mediators between the two. This suggests that improving the subjective social status of older adults, increasing the frequency of their media use, and enhancing their wellbeing can enhance the health-promoting effects of social networks.

Furthermore, this study finds that labor participation can improve older adult health, aligning with the research of Staudinger et al. (48) and Di Gessa et al. (49). The moderating effect test indicates that labor participation negatively moderates the impact of social networks on older adult health, weakening the relationship between the two. This implies that labor participation can substitute the positive effects of social networks on older adult health. The reason for this substitution effect may lie in the fact that engaging in work brings positive emotions such as tranquility, inspiration, and happiness (50) as well as a more stable income, whereas the health-promoting effects of social networks also encompass increased positive emotions (i.e., wellbeing) (44) and economic status (i.e., social class).

Further investigation reveals that the substitution effect of agricultural labor is not significant, whereas non-agricultural labor participation can partly substitute social networks in promoting older adult health, which is consistent with the findings of Lee and Kim (51). In China, agricultural labor typically implies low wages, and low-wage work has adverse health effects (52). Compared to agricultural work, non-agricultural labor is less physically demanding, leading to fewer health-related drawbacks and more favorable outcomes for older adult health.

Research on heterogeneity shows that, in terms of physical health, the impact of social networks on both physical and mental health is more pronounced for men. This may be attributed to the traditional Chinese division of labor, where women primarily engage in family-oriented activities, often centering their social networks around their husbands. Consequently, the influence of familial ties on women is less significant compared to men, resulting in a lower density of effective social networks for women and a diminished positive effect of these networks on health.

Regarding physical health, the influence of social networks on the health of older adults with varying educational backgrounds is significant; however, the coefficients for those with higher education levels are greater, indicating that older adults with higher education are more likely to benefit from social networks in promoting their physical health. This may be because older adults with higher education are often situated within networks of greater overall quality, tend to share health knowledge, and maintain healthy habits, making it easier for them to align with others in their network, thereby enhancing their physical health.

In terms of mental health, social networks significantly improve the psychological wellbeing of older adults with lower education levels, but the impact on those with higher education is not statistically significant. This may be due to lower-educated individuals obtaining diverse information through social networks, facilitating communication and mutual support, thereby counteracting the erosion of social roles and promoting mental health. In contrast, higher-educated older adults, while benefiting from similar effects, may also experience peer pressure from their networks, leading to anxiety that undermines their mental health. The combined influence of these factors renders the effect of social networks on the mental health of higher-educated older adults insignificant.

In addition, this study comprehensively considers issues of internal validity and generalizability. To address the issue of internal validity, the study reduces the risk of omitted variable bias by incorporating a full set of control variables and conducts robustness tests using a variety of approaches to confirm the stability of the results. This study utilizes data from the 2021 China General Social Survey (CGSS), which is representative of China’s older adult population. The inclusion of different subgroups (of different ages and educational attainment) in the study allows for broader applicability across different demographic and policy contexts.

Based on the findings, several policy recommendations can be proposed.

First, strengthening the Social Support Network for the Older Adult. First, it is essential to advocate for the establishment of harmonious intergenerational relationships within families and to extend family leave policies to promote positive interactions between older adult individuals and their children or relatives. This can leverage strong relational ties to enhance the health and wellbeing of the older adult. Additionally, there should be efforts to create an environment conducive to older adult social participation by improving public service facilities and constructing age-friendly communities. The social networks comprising family members, neighbors, communities, and social organizations should be harnessed to build a robust support system that enables bottom-up, targeted services for the older adult.

Second, enhancing Age-Friendly Media Services. Addressing the issue of digital exclusion among the older adult is crucial. This can be achieved by improving age-appropriate standards and guidelines for digital technologies and expanding the provision of age-friendly smart devices tailored to older adult individuals’ daily activities and usage scenarios. It is also vital to guide the older adult in effectively using the Internet and mobile social media platforms. A comprehensive and targeted new media service strategy should be developed to mitigate the digital integration challenges faced by the older adult.

Third, mitigating Social Stratification. A comprehensive social security system should be established, along with an optimized resource distribution mechanism, to integrate urban and rural pension schemes and enhance the pension benefits for all residents. This would ensure that the older adult can equally share the benefits of economic development. Additionally, diversified older adult care services should be developed to meet the varied needs of the rural older adult population, thereby addressing health inequities caused by social stratification among the older adult.

Fourth, improving the wellbeing of the older adult. From a demand-side perspective, efforts should be made to enhance the older adult care system, continuously refine the basic older adult care service catalog, and promote the balanced and high-quality development of older adult care services. This would enable all older adult individuals to access basic care services more effectively. Additionally, it is essential to encourage the older adult to adopt a positive outlook on aging by integrating the concepts of “active aging” and “healthy aging” into all aspects of socio-economic development. This approach aims to enhance the older adult’s sense of happiness, satisfaction, and security.

Fifth, promoting Older Adult Labor Participation. The positive effects of labor participation on health should be widely communicated, while creating a supportive environment for older adult reemployment. Social organizations and enterprises should be encouraged to provide skill training and employment services for older adult individuals who wish to reenter the workforce. Furthermore, the potential of older adult individuals should be actively tapped by encouraging their involvement in cultural practices, volunteer services, and other non-agricultural activities according to their circumstances. This would foster a mindset of active aging, enabling the older adult to realize their value and contribute meaningfully to society.

Given the limitations in time and resources, the sample selected for this study is limited. The representativeness and typicality of the sample exhibit some deficiencies and require further refinement in future research. (1) While defining health levels through inquiries about the physical and mental health of older adults provides a degree of representativeness and reflects their real circumstances to some extent, it remains somewhat subjective. Future studies should employ standardized measures such as the Activity of Daily Living Scale (ADL) and the Kessler 6 Rating Scale (K6) to assess the health levels of older adults and to re-analyze and validate conclusions. (2) The research model employed in this study is relatively simple; future work should consider refining and optimizing the research model and methodology to enhance the persuasiveness of the findings, thereby providing more comprehensive and effective recommendations for improving the health levels of older adults.

## 7 Conclusion

First, social networks exert a significant positive influence on the health of older adults, with weak ties being more closely associated with health outcomes. The sub-sample regression results indicate that social networks have a more pronounced impact on the physical health of men and highly educated older adults, while the mental health of men and lower-educated older adults is also more significantly affected. Second, media usage, subjective social status, and wellbeing serve as crucial explanatory mechanisms for the influence of social networks on the health of older adults. Notably, subjective social status and wellbeing of older adults play a chain-mediated role in the relationship between social networks and both physical and mental health. Third, active labor participation represents a viable option for older adults to enhance their health levels, with labor participation being able to substitute the benefits provided by social networks. Moreover, the substitution effect of non-agricultural labor participation is more pronounced compared to that of agricultural participation.

## Data Availability

The original contributions presented in the study are included in the article/supplementary material, further inquiries can be directed to the corresponding author.
